# Draft genome sequence of a steroid-transforming actinomycete isolated from African clawed frog *Xenopus laevis*

**DOI:** 10.1128/mra.00709-25

**Published:** 2025-10-22

**Authors:** Vyacheslav V. Kollerov, Eugeny Y. Bragin, Marina V. Donova

**Affiliations:** 1 Federal Research Center, Pushchino Center for Biological Research of the Russian Academy of Sciences, G.K. Skryabin Institute of Biochemistry and Physiology of Microorganisms, Pushchino, Moscow region, Russia; California State University San Marcos, San Marcos, California, USA

**Keywords:** actinomycete, steroid-transforming, genome sequence, *Xenopus laevis*, frog

## Abstract

This study presents the draft genome sequence of a steroid-transforming actinomycete isolated from the *Xenopus laevis* frog. Based on the 16S rRNA gene sequence and the DNA-DNA hybridization *in silico*, the isolated strain was identified as *Streptomyces rochei* MTOC-St3. The data obtained expand knowledge of the diversity of steroid-transforming actinomycetes inhabiting amphibians.

## ANNOUNCEMENT

Mucous integuments of amphibians, such as frogs, are known to be inhabited by various symbiotic bacteria that play a vital role in protecting the host organism against pathogens (1–3).

Earlier, a culture of actinomycete was isolated from the dermal mucosa of African clawed frog *Xenopus laevis* according to the previously described protocol (https://www.protocols.io/view/isolation-of-microbes-from-the-skin-of-terrestrial-kxygx35zzg8j/v1) (4) and kindly provided by Dr. Suzina (Pushchino Center for Biological Research of the Russian Academy of Sciences). Preliminary studies revealed a steroid-transforming activity of the microbial isolate toward 17α,21-dihydroxypregn-4-ene-3,20-dione. Using mass spectrometry and ^1^H NMR spectroscopy, 17α,20β,21-trihydroxy-4-pregnen-3-one has been identified as a major metabolite, thus indicating the presence of a 20β-reducing activity.

The isolate was maintained on the SMY agar medium: potato starch—10 g/L; malt extract—10 g/L; yeast extract—4 g/L; agar—15 g/L; and pH 7.0. The spore suspension obtained from the SMY agar slant was inoculated into 50 mL of the liquid SMY medium and incubated aerobically in 750 mL Erlenmeyer flasks on a rotary shaker (200 rpm) at 30°C for 24 h. The inoculum (10% v/v) was transferred to 45 mL of fresh SMY medium and incubated under the same conditions. After 18 h of growth, the cells were pelleted by centrifugation at 1800 × *g* and 4°C for 10 min. Genomic DNA was isolated from cells using the DNeasy Blood and Tissue Kit (Qiagen, USA) according to the manufacturer’s instructions. The library was prepared using the Illumina DNA Prep, (M) Tagmentation Kit and sequenced on Illumina NovaSeq 6000 (Illumina, San Diego, CA, USA) to generate 7,991,849 reads 2 × 150 bp long. The demultiplexing of the sequencing reads was performed with Illumina bcl2fastq (version 2.20). Default parameters were used for any software, unless otherwise specified. Adapters were trimmed with Skewer (version 0.2.2) (5). The *de novo* genome assembly and its annotation were performed using the Ray 1.1.1 (6) and PGAP 6.8. programs (7) correspondingly. The results of the genome assembly and annotation are presented in Table 1.

**TABLE 1:** Results of genome assembly, annotation, and calculation of DDH and ANI

General sequencing results					
Number of contigs	Total length of all contigs, bp	Value of parameter N50	Protein-coding genes	RNA-coding genes	Pseudogenes
567	9,156,005	93,562	8,236	90	129

Comparison of the sequenced genome of *Streptomyces rochei* MTOC-St3 with the genome of a typical strain *Streptomyces rochei* NRRL B-2410				
DDH (formula: DDH = HSP length/total length)	DDH (formula: DDH = identities/HSP length)	DDH (formula: DDH = identities/total length)	ANIb (%)	ANIm (%)
88.5	90.6	91.5	98.07	98.94

A gene was found in the genome of this strain whose product is 60.5% similar to cholesterol oxidase ChoD of *Mycobacterium tuberculosis* (CAB01014.1). No other genes of the main sterol utilization pathway were identified.

The preliminary identification of the strain was based on the 16S rRNA gene sequence. The 16S rRNA gene fragment was amplified with universal primer pair 27F and 1492R and Q5 high-fidelity DNA polymerase (New England BioLabs) and sequenced by using the 3500 Series Genetic Analyzer (Thermo Fisher Scientific, USA). The sequence homology search performed by the BLAST+ 2.16.0 (dbV5) showed that this gene fragment has the highest identity (99.86%) with the sequence of the 16S rRNA gene fragment from *Streptomyces rochei* 173831 (EU570561.1).

To confirm the species identity, the DNA-DNA hybridization (DDH) *in silico* and the calculation of the average nucleotide identity (ANI) were performed between the studied strain and the genome of the type strain *Streptomyces rochei* NRRL B-2410 (MUMD00000000.1). DDH was carried out using the Genome-to-Genome Distance Calculator 3.0 (GGDC 3.0) program (8), and ANI calculation was made with the JspeciesWS online service (9). Both the DDH and the ANI indicate that the isolate belongs to the species *S. rochei* (Table 1).

## Data Availability

The draft genome assembly has been deposited in GenBank under accession no. JBIENY000000000.1. The raw reads have been deposited to the NCBI Sequence Read Archive under accession no. SRX25990076.
